# Autophagy in Plants: Both a Puppet and a Puppet Master of Sugars

**DOI:** 10.3389/fpls.2019.00014

**Published:** 2019-01-22

**Authors:** Henry Christopher Janse van Rensburg, Wim Van den Ende, Santiago Signorelli

**Affiliations:** ^1^Laboratory of Molecular Plant Biology, KU Leuven, Leuven, Belgium; ^2^Departamento de Biologiía Vegetal, Facultad de Agronomía, Universidad de la Repuíblica, Montevideo, Uruguay

**Keywords:** stress, autophagy, sugar, SnRK1, target of rapamycin, ABA

## Abstract

Autophagy is a major pathway that recycles cellular components in eukaryotic cells both under stressed and non-stressed conditions. Sugars participate both metabolically and as signaling molecules in development and response to various environmental and nutritional conditions. It is therefore essential to maintain metabolic homeostasis of sugars during non-stressed conditions in cells, not only to provide energy, but also to ensure effective signaling when exposed to stress. In both plants and animals, autophagy is activated by the energy sensor SnRK1/AMPK and inhibited by TOR kinase. SnRK1/AMPK and TOR kinases are both important regulators of cellular metabolism and are controlled to a large extent by the availability of sugars and sugar-phosphates in plants whereas in animals AMP/ATP indirectly translate sugar status. In plants, during nutrient and sugar deficiency, SnRK1 is activated, and TOR is inhibited to allow activation of autophagy which in turn recycles cellular components in an attempt to provide stress relief. Autophagy is thus indirectly regulated by the nutrient/sugar status of cells, but also regulates the level of nutrients/sugars by recycling cellular components. In both plants and animals sugars such as trehalose induce autophagy and in animals this is independent of the TOR pathway. The glucose-activated G-protein signaling pathway has also been demonstrated to activate autophagy, although the exact mechanism is not completely clear. This mini-review will focus on the interplay between sugar signaling and autophagy.

## Introduction

Autophagy is a mechanism by which eukaryotic cells transport cellular components to lytic vacuoles where they are degraded and recycled. Basal autophagy is maintained under non-stressed conditions for cellular homeostasis, but the intensity of autophagy is typically further increased under stress to provide temporal stress relief (Inoue et al., 2006; Yang and Bassham, 2015). Macro-autophagy (stress-induced autophagy, or in short autophagy) involves the delivery of undesirable cytoplasmic materials by specialized double-membrane vesicles (autophagosomes) to the lytic compartment for their removal and/or to provide energy and building blocks for cellular processes (Li and Vierstra, 2012; Liu and Bassham, 2012; Yoshimoto, 2012). More specific modes of autophagy include the specific recycling of organelles and specific proteins (Svenning et al., 2011; Floyd et al., 2012; Schreiber and Peter, 2014).

The process of autophagosome formation is described in detail elsewhere (Lamb et al., 2013). The final step includes the fusion of the autophagosome membrane with the lytic compartments (vacuoles in plants and yeast, lysosomes in animals). The whole process relies on more than 30 AuTophaGy-related (ATG) genes (for a detailed review see Mizushima et al., 2011). The ATG proteins were initially identified in yeast, but their orthologs are highly conserved in eukaryotes (Tsukada and Ohsumi, 1993; Mizushima et al., 2011; Yu et al., 2018).

Autophagy is closely associated with the metabolic status of cells, and its regulation should closely link to sugar signaling and sensing mechanisms, especially under stress. This review will discuss the current understanding of autophagy in plants under stressed and non-stressed conditions, with focus on the role of sugars and sugar signaling pathways in the process. In particular, we will discriminate between “sugar starvation” and “sugar excess” types of autophagic responses.

It should be noted that some stresses (e.g., extended darkness, acute heat stress) induce sugar starvation responses (Slocombe et al., 2004; Barros et al., 2017) while other stresses (e.g., slowly progressing drought, salt and cold stresses) lead to sugar excess (Krasensky and Jonak, 2012; Tarkowski and Van den Ende, 2015).

## The Function of Autophagy in Plants Under Non-Stressed Conditions

Initially autophagy was thought to be a non-specific bulk removal and transport of cytoplasmic material to lytic vacuoles where the content is recycled, but it became clear that it is a tightly regulated and much more specific process, controlling overall plant development, metabolism, senescence, biotic and abiotic stress responses, and innate immunity (Liu and Bassham, 2012; Wang et al., 2017). Although basal autophagy contributes to cellular homeostasis during growth, the majority of *atg* mutants complete their life cycle without detrimental defects (Doelling et al., 2002; Thompson et al., 2005; Phillips et al., 2008). In plants with suppressed autophagy, however, general fitness is compromised, including reduced growth, early leaf senescence, altered anthocyanin levels and hypersensitivity to several stresses (Masclaux-Daubresse et al., 2014, 2017; Wang et al., 2017; Bárány et al., 2018; Jiménez-Nopala et al., 2018; Minina et al., 2018). In contrast, plants over-expressing autophagy genes show increased resistance to necrotrophic pathogens and oxidative stress, enhanced growth and delayed aging (Minina et al., 2018). For an extensive overview of the mechanisms and proteins investigated on autophagy to date see the review by Yoshimoto and Ohsumi (2018).

Focusing on seed development, autophagy has been linked to seed maturation in maize following pollination, by increasing the lipidation of the ATG8 protein in the endosperm (Chung et al., 2009). This was also the case after seed germination, illustrating that autophagy plays a role in the remobilization of nutrients from the endosperm to support early seedling development (Chung et al., 2009). Abscisic acid (ABA) and ethylene are necessary for basic development and were linked to basal autophagy (Yu and Xie, 2017; Ceusters and Van de Poel, 2018). Autophagy has also been linked to regulating the supply of nutrients during the development of anthers in rice (Zhang et al., 2011; Kurusu et al., 2014). It is believed that autophagy regulates the supply of nutrients in the tapetum cells of monocots, and rice autophagy defective mutants are male sterile due to a lack of lipid and starch accumulation in pollen grains (Kurusu et al., 2014). Dicots produce lipidic tapetosomes, whereas monocots do not form the tapetosomes required for transport of lipids in tapetal cells. Autophagy seems to play a role in postmeiotic anther development through degradative processes in tapetum cells. Thus the dicot Arabidopsis autophagy mutants do not share this defect (Kurusu et al., 2014). UDP-Glucose (UDP-Glc) was recently proposed as a potential signaling molecule and regulator of autophagy in plants (Janse van Rensburg and Van den Ende, 2017). This was suggested on the basis of Arabidopsis UDP-glucose pyrophosphorylase (UGPase) mutants with reduced UDP-Glc showing severe vegetative growth defects and male sterility, which could be rescued by exogenous UDP-Glc application but not by Sucrose (Suc) (Park et al., 2010). Interestingly, Arabidopsis Suc synthase (SuSy) mutants with reduced Suc breakdown (lower UDP-Glc) in seeds showed decreased starch in the seed coat and it was suggested that starch synthesis is regulated by the downstream metabolites rather than by SuSy itself (Angeles-Núñez and Tiessen, 2010, 2012). In contrast, rice mutants accumulating UDP-Glc developed spontaneous programmed cell death (PCD), a phenotype also observed in seedlings treated with exogenous UDP-Glc (Xiao et al., 2018). Autophagy may contribute to the PCD phenotype observed in plants with increased UDP-Glc, potentially signaling metabolic imbalances.

## Sugar Starvation Autophagy and the SnRk1/Tor Nexus

During nutrient starvation, autophagy helps to recycle metabolites. This is evident from *ATG* gene expression studies and the reactions of *atg* mutants exposed to carbon and nitrogen starvation (Thompson et al., 2005; Avila-Ospina et al., 2014; Mukae et al., 2015; Soto-Burgos and Bassham, 2017; Di Berardino et al., 2018; Sun et al., 2018). During nitrogen starvation, *atg* mutants display a hypersensitive response (HR) with reduced remobilization and seed production compared to wild-type (WT) plants, indicating that autophagy assists in nitrogen remobilization (Guiboileau et al., 2012; Wada et al., 2015). Besides its role during nitrogen recycling, autophagy also plays an important role during starch remobilization (Izumi et al., 2013a,b; Wang et al., 2013; Wada et al., 2015). Under carbon starvation, growth of *atg* mutants is reduced due to the accumulation of proteins and reduced amino acid levels (Di Berardino et al., 2018). Autophagy mutants also show a reduction in amino acid synthesis during carbon starvation, indicating its major contribution to maintain cellular homeostasis (Izumi et al., 2013a; Avin-Wittenberg et al., 2015).

How are *ATG* genes regulated in plants? Historically, plant research is running behind on animal and yeast research. It was found that AMPK (animals) and Snf-1 (yeast) are important energy and metabolic sensors regulating cellular homeostasis in balance with the TOR-kinase complex, and both kinases clearly link to autophagy (Noda and Ohsumi, 1998; Pattingre et al., 2008; Liu and Bassham, 2010). SnRK1 is the plant ortholog of the AMPK and Snf-1 proteins (Sugden et al., 1999; Crozet et al., 2014). The interplay of SnRK1 and TOR is often referred to as the “Yin and Yang” of controlling metabolites and biological processes of cells in response to metabolic and environmental stimuli (Dobrenel et al., 2016). During low energy and nutrient starvation, AMPK and Snf1 inhibit anabolic processes such as protein, fatty acid and cholesterol synthesis, whereas catabolic processes such as glycolysis, fatty acid oxidation and autophagy are activated (Crozet et al., 2014). The AMPK/Snf1 complex in animals and yeast regulates autophagy via at least two pathways, the first being through inhibiting TOR (Lee et al., 2010), thus preventing inhibition of autophagy, and secondly by directly phosphorylating ATG1, which activates autophagy (Wang et al., 2001; Egan et al., 2011; Kim J. et al., 2011).

**Figure 1:**
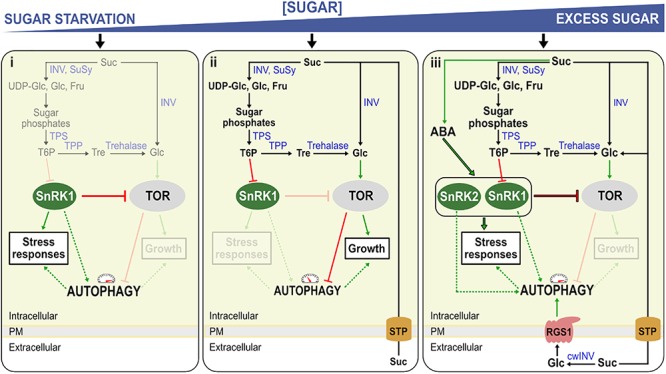
Model for the regulation of autophagy in Arabidopsis seedlings under sugar starvation and sugar excess. The model represents three conditions, **(i)** sugar starvation, **(ii)** normal (unstressed) growth conditions, **(iii)** high sugar levels and/or stress conditions. **(i)** Low T6P levels activate sugar starvation-based autophagy, by rendering SnRK1 active. Active SnRK1 in turn inhibits TOR, activating autophagy. **(ii)** Suc is broken down to sugars and sugar phosphates by Suc synthase (SuSy) and invertases (INV). When these Suc-splitting enzymes (Susy/INV) are readily able to deal with Suc, T6P is synthesized by TPS and TPP, its level mirroring Suc levels. T6P inhibits SnRK1 which on its turn inhibits TOR-kinase, an inhibitor of autophagy. **(iii)** Excess sugar levels (excessive import, exogenous sugar supply) can probably also induce autophagy, at least partly through stimulating ABA synthesis and signaling the SnRK2/TOR nexus. Suc can also be metabolized in the apoplast by cell wall INV (cwINV), producing free Glc and Fru. Normally, these hexoses are rapidly imported by sugar transport proteins (STP) and used for growth associated with TOR signaling. Under stress, growth is compromised, leading to reduced uptake and increased extracellular Glc which can be sensed by the Regulator of G-protein signaling (RGS1), in turn activating autophagy through an unknown mechanism. Red blunt arrows indicate negative regulation and green arrows positive regulation. Dashed lines represent potential regulatory mechanisms. Enzymes are indicated in blue. Mechanisms that are not active during a specific condition are faded.

In contrast to its animal and yeast counterparts, SnRK1 uses small phosphorylated sugars [glucose-6-phosphate (Glc6P), glucose-1-phosphate (Glc1P), trehalose-6-phosphate (T6P)] as the gauge of cellular energy status instead of directly by AMP (Figure 1; Broeckx et al., 2016 and references therein). Most focus is on the sugar phosphate T6P (Figure 1), signaling Suc availability, but also functioning as a negative feedback regulator of Suc levels, contributing to cellular Suc homeostasis (Figueroa and Lunn, 2016). In this regard, T6P acts as a negative regulator of SnRK1 through direct interaction with the catalytic subunit, KIN10, thus translating cellular Suc status (Zhai et al., 2018). In response to starvation, SnRK1 activates several downstream components such as the basic leucine zippers (bZIPs), specifically the G-box binding factor (GBF5), bZIP11, and bZIP63 (Baena-González et al., 2007; Delatte et al., 2011; Mair et al., 2015). These transcription factors control the expression of genes involved in catabolic pathways such as the degradation of cell walls, amino acids, protein, starch and initiation of autophagy to provide alternative sources of metabolites and energy under sugar starvation conditions (Baena-González et al., 2007). Similar to the situation in animals, the KIN10 catalytic subunit phosphorylates the regulatory-associated protein of mTOR (RAPTOR) from the TOR complex (Nukarinen et al., 2016).

SnRK1 is a heterotrimeric complex (Sugden et al., 1999; Crozet et al., 2014). The SnRK1 complex consists of a catalytic/kinase (α) and two regulatory (β, γ) subunits (Crozet et al., 2014). The regulatory subunits are classified into two groups, the plant specific subunits (β3 and βγ), and the classical subunits (β1, β2, and γ) which are conserved between mammals and yeast (Halford et al., 2003; Emanuelle et al., 2015; Broeckx et al., 2016). In animals the γ subunit functions as the sensor for energy (adenylate binding). It is interesting that the majority of the active SnRK1 complexes in plants consist of a βγ hybrid subunit that acts as the canonical γ subunit, even though plants have a specific γ subunit (Ramon et al., 2013). Arabidopsis has three catalytic subunits of SnRK1, namely KIN10, KIN11, and KIN12, however, it seems that only KIN10 and KIN11 are expressed in vegetative tissues and the majority of SnRK1 can be attributed to KIN10 (Baena-González et al., 2007; Jossier et al., 2009; Wurzinger et al., 2017). When KIN10 and KIN11 are overexpressed, plants show either late or early flowering, and *kin10kin11* double mutants appear to be lethal, suggesting potential redundancy (Baena-González et al., 2007; Williams et al., 2014). Single mutants of *kin10* and *kin11* have the WT phenotype, however, reduced expression of both genes causes several developmental defects and lower responses of stress-and-starvation-related genes (Baena-González et al., 2007).

TOR is a negative regulator of autophagy, probably through the conserved ATG1/ATG13 kinase inhibition (Suttangkakul et al., 2011). In Arabidopsis, the TOR complex forms the major component of the TOR signaling pathway and it consists of three main components, the serine/threonine kinase TOR (Menand et al., 2002), RAPTOR (Anderson et al., 2005; Deprost et al., 2005), providing the substrates for phosphorylation by TOR (Hara et al., 2002), and LST8, the stabilizer of the complex (Moreau et al., 2012). For an in-depth review of the TOR complex in plants see Schepetilnikov and Ryabova (2018). TOR is expressed at high levels in actively growing Arabidopsis tissues such as endosperm, meristem and embryo (Menand et al., 2002). Plants with reduced TOR expression showed stunted root growth, whereas over-expressing plants showed enhanced root growth (Deprost et al., 2007). In general, TOR is activated in nutrient-rich conditions stimulating growth, and in sink tissues, in particular, by Glc derived from imported Suc (Figure 1ii; Xiong and Sheen, 2012, 2013; Xiong et al., 2013). TOR regulates autophagy alongside other growth-promoting processes such as the initiation of translation in response to nutrient availability (Deprost et al., 2007; Xiong and Sheen, 2015). In animals, the TOR complex prevents ATG13-ULK1 interaction by directly phosphorylating ATG13, thus inhibiting autophagy, whereas AMPK promotes autophagy by the direct phosphorylation of ULK1 (Kim et al., 2011). The activator of autophagy, ULK1, is the animal homolog of the serine/threonine kinase ATG1 in plants. Alternatively, AMPK in animals can phosphorylate the TOR complex, rendering autophagy active (Gwinn et al., 2008). In plants, it is not completely clear whether SnRK1 and/or TOR can directly phosphorylate ATG1 (as is the case for ULK1 in animals), urging further research in this area (Suttangkakul et al., 2011; Chen et al., 2017). In any case, when the catalytic subunit KIN10 is over-expressed in plants, phosphorylation of ATG1 increases (Chen et al., 2017), and SnRK1 and ATG1 interaction seems to be present in all tissue types (Chen et al., 2017).

Arabidopsis plants with disrupted TOR-kinase expression showed reduced growth due to constitutive autophagy, whereas a complete knockout of TOR is embryo-lethal (Menand et al., 2002; Deprost et al., 2007; Liu and Bassham, 2010). Nevertheless, overexpression of TOR prevents autophagy activation during several abiotic stresses (Pu et al., 2017). Interestingly, constitutive TOR expression inhibited autophagy even in plants over-expressing SnRK1 during stress conditions, illustrating that TOR plays a central regulatory role during autophagy, acting downstream of SnRK1/AMPK, both in plants and animals (Pu et al., 2017).

KIN10 activates autophagy by inhibiting the TOR signaling pathway (Soto-Burgos and Bassham, 2017). When TOR is inhibited, autophagy is activated, and the inhibition of SnRK1 has no effect on this activation. Increased SnRK1 activity does not induce autophagy when TOR is still active, confirming that SnRK1 acts upstream of TOR (Soto-Burgos and Bassham, 2017). Thus, SnRK1 can induce autophagy both via TOR-dependent and TOR-independent pathways in Arabidopsis. In concordance, plants over-expressing KIN10 exhibited a typical “sugar starvation” type of autophagy, including an increased adaptation to nutrient starvation, increased abiotic stress tolerance and delayed leaf senescence (Baena-González et al., 2007; Li et al., 2014).

## The Potential Role of the SnRk2/Tor Nexus in Sugar Excess Autophagy

So far, most of the research focused on “sugar starvation” mediated autophagy. Yet, accumulating evidence suggest that autophagy can also intensify under “sugar excess” conditions. In animals for instance, diabetes (increased Glc levels in the blood) promote autophagy (Moruno et al., 2012) and progressive loss of cardiac cells (Munasinghe et al., 2016). Likewise, the most devastating abiotic stresses in plants (drought, salt and cold stresses) typically lead to increased sugar levels in leaves due to disturbed source-sink balances (Krasensky and Jonak, 2012). In the resurrection species *Tripogon loliiformis*, increased Suc and trehalose (Tre) levels coincided with autophagosome formation (Williams et al., 2015). Moreover, autophagy is induced by salt stress and was demonstrated to be essential for ABA-mediated salt tolerance (Luo et al., 2017). Under stress, ABA-activated SnRK2s phosphorylate RAPTOR (Wang et al., 2018). Thus, it is tempting to speculate that ABA and sugar excess would stimulate autophagy mainly through the SnRK2/TOR nexus, although ABA was reported to also stimulate SnRK1 (Rodrigues et al., 2013; Figure 1iii). High sugar and ABA trigger natural leaf senescence (Pourtau et al., 2006; Gao et al., 2016). The triple mutant *snrk2.2/2.3/2.6* exhibited a stay-green phenotype after ABA treatment (Gao et al., 2016). ABA, which is known to be systemically induced upon several stress conditions, induces TOR inhibition through SnRK2s, allowing autophagy to take place independently of the energetic cellular level (Figure 1iii). High levels of Suc may enhance ABA signaling (Huijser et al., 2000; Rook et al., 2002) and SnRK2-mediated TOR inhibition (Figure 1iii). Suc and its non-metabolizable analog, turanose, were shown to induce ABA accumulation in strawberry fruits (Jia et al., 2013), suggesting that Suc signaling may boost ABA synthesis and signaling (Figure 1iii), but this connection needs further exploration. Interestingly, Suc and its non-metabolizable analog palatinose induced AGPase activation in potato tubers through SnRK1 (Tiessen et al., 2003).

## Extracellular Glc, G Protein Signaling, and Autophagy

**Figure 2:**
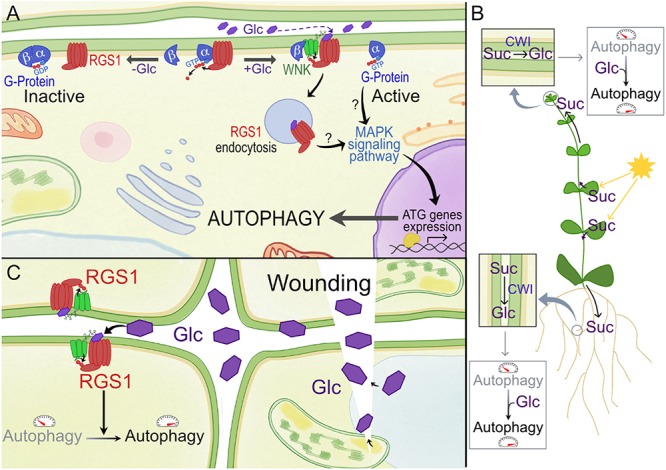
Model for activation of RGS1 by glucose (Glc) and its potential effect on autophagy in plants. **(A)** In plants, the Gα subunit spontaneously exchanges GDP, and 7-transmembrane RGS proteins such as RGS1 maintain the inactive state of G protein signaling. GPA1 (Gα subunit of G-protein) binds both RGS1 and its partner AGB1/AGG (Gβ). Upon extracellular Glc binding, it is proposed that the equilibrium of Gα binding shifts from Gβ to RGS. Free Gβ then recruits WNK kinase for RGS1 phosphorylation and internalization. Upon internalization, G protein signaling is self-activated and sustained. Internalization of RGS1 occurs in correlation with autophagic pathways, potentially through MAPK signaling pathways. **(B)** The most active photosynthetic leaves produce Suc to feed the sink tissues. After apoplastic unloading, this Suc can be converted to hexoses by CWI, and the released Glc moieties, when not immediately imported and metabolized into cells, may surpass a certain threshold level in the apoplast, triggering extracellular sugar signaling mediated by RGS1. Autophagy is known to be more active in developing sink tissues. In this way, Glc can contribute to promote autophagy in these tissues. **(C)** Wounding due to abiotic or biotic stress can result in the sudden increase of apoplastic Glc. This DAMP signaling can lead to the induction of autophagy in neighboring cells, which is known to be relevant for (a)biotic stress tolerance.

Plasma membrane (PM) receptors perceive apoplastic signals. Heterotrimeric G protein complexes transfer the extracellular signal to the intracellular environment (Figure 2A). Upon activation, the heterotrimeric G protein, located at the cytoplasmic side of the membrane, exchanges GDP for GTP (Urano et al., 2013; Urano and Jones, 2014). The GTP bound complex can then interact with intracellular components (Kleuss et al., 1994; Chuang et al., 1998; Urano et al., 2012). Plants have 7-transmembrane regulator of G-protein signaling (RGS) proteins that maintain the inactive state of the complex (Jones et al., 2011), and G signaling is activated when the receptor-RGS protein is internalized through endocytosis (Figure 2A). In the resting state, AtRGS1 binds to the Gα subunit, AtGPA1, thus maintaining the inactive state of G protein signaling. Although still under debate, it is assumed that Glc activates G-protein signaling through the RGS1 receptor by binding directly to the extracellular 7-transmembrane region of RGS1 (Grigston et al., 2008). Increasing levels of extracellular Glc recruits a with-no-lysine kinase (AtWNK) that phosphorylates RGS, which leads to the endocytosis of RGS1 (Figure 2A; Urano et al., 2012; Fu et al., 2014).

A few studies in animals also point to a relationship between the autophagy pathway and RGS (Ogier-Denis et al., 2000; Pattingre et al., 2003; Garcia-Marcos et al., 2011; Law et al., 2016). Recently, endocytosis of RGS1 has been linked to autophagy pathways in plants (Yan et al., 2017). It was demonstrated that autophagy plays an essential role in regulating the Glc-induced RGS1-mediated signaling pathway in Arabidopsis (Figure 2A). Autophagy not only promoted the endocytosis of RGS1, but also inhibited its recovery to the membrane during Glc treatment (Yan et al., 2017). The expression of several *ATG* genes was also up-regulated in response to Glc treatments in WT plants but not in RGS1 null mutants, indicating that extracellular Glc induces autophagy via RGS1 (Yan et al., 2017). The interplay between autophagic and endocytotic pathways is well known in plants (Pečenková et al., 2017; Zhu et al., 2018). This interplay should regulate either the recycling of RGS1 back to the PM or degradation in the vacuole. It is not yet clear whether RGS1 is actually localized within the autophagic body after Glc-mediated endocytosis (Yan et al., 2017). It can be debated that RGS1 activates autophagy via the G-protein signaling pathway, and this in turn recycles the endocytosed RGS1 to the vacuole and that *de novo* synthesized RGS1 is then re-located to the PM. It should be noted that this is in contradiction with photosynthetically derived Glc which activates TOR, thus repressing autophagy, suggesting compartmentalization differences (Xiong et al., 2013).

Strongly increased extracellular Glc signals may be perceived as “danger,” and extracellular Glc may be considered as a damage-associated molecular pattern (DAMP) candidate (Versluys et al., 2016 and references therein). Focusing on sink tissues, where the roles of the putative RGS1 Glc sensor and cell wall invertase (CWI) are best understood, apoplastic Glc levels depend on the balance between Suc unloading (depending on photosynthesis and leaf export efficiency), CWI activity and import efficiency of Glc into the cells (Figures 1iii, 2B). It should be noted that CWI and vacuolar invertase (VI) are mainly regulated at the post-translational level by invertase inhibitors (Hothorn et al., 2010). Evidently, increased apoplastic Glc can also originate from cellular leakage processes under stress (Figure 2C). In this regard, increased extracellular Glc levels, above certain threshold levels, may be involved in inducing autophagy (Yan et al., 2017; Figures 1iii, 2B). Autophagy was reported to be more active in developing sink tissues, in particular during seedling growth (Kim et al., 2013) and during cellular architectural remodeling required under differentiation and development (Bassham et al., 2006). RGS1 mutants develop etiolated hypocotyls partially due to Glc insensitivity (Chen et al., 2006; Huang et al., 2015). G protein signaling is known to promote seedling elongation through activation of the cell cycle (Ullah et al., 2001; Ullah, 2003; Chen et al., 2006). Thus, autophagy seems to be key in seedling establishment, plant development and reproduction, potentially through RGS1 (Figure 2B).

## Autophagy and Ros Homeostasis Under Oxidative Stress

During oxidative stress, the production of reactive oxygen species (ROS) by respiratory burst oxidase homolog (Rboh), acts as the signal for the activation of stress responses, including autophagy (Wang et al., 2017). Autophagy is regulated through both Rboh-dependent and -independent pathways (Liu et al., 2009; Chen et al., 2015). Arabidopsis *atg* mutants are hypersensitive to submergence-induced hypoxia, linked to salicylic acid-signaling pathways (Chen et al., 2015). In animals, autophagy contributes to cell survival during hypoxia (Kroemer et al., 2010). In plants, most abiotic stresses including hypoxia submergence lead to oxidative stress through ROS increases. Under oxidative stress, SnRK1 and AMPK activate autophagy (Rabinovitch et al., 2017; Soto-Burgos and Bassham, 2017), helping organisms to overcome these stresses. However, oxygen deprivation during hypoxia also leads to a switch to anaerobic respiration, thus a decrease in energy produced which can directly activate autophagy through the SnRK1/TOR pathway (Voesenek and Bailey-Serres, 2013; Soto-Burgos and Bassham, 2017). ROS production by Rboh is necessary for plant tolerance to submergence and activation of autophagy (Chen et al., 2015). However, ROS may oxidize key proteins in these signaling pathways, threatening response viability. For example, in mammals, TOR is known to be oxidized and inactivated by H_2_O_2_, and a specific thioredoxin directly interacts with TOR to prevent its oxidation and ensure its functionality (Oka et al., 2017). Thus, the oxidative status and the activity of the SnRK1/TOR nexus are expected to be crucial during autophagic responses, linking cellular sugar and ROS homeostasis. Deeper studies are warranted in this area, under different environmental conditions, since the composition and concentration of sugars as well as ROS species can greatly vary under these conditions.

## Links Between Alternative Sugars and Autophagy

Autophagy itself also regulates sugar levels. In animal cells, exogenous Tre, Suc and raffinose induce autophagy independent of TOR (Chen et al., 2016). It is proposed that after a certain amount of uptake, autophagy is induced in an attempt to aid in the breakdown of these sugars. Considering that animal cells do not usually contain high levels of Suc or raffinose, this points to a mechanism employed to remove accumulating sugars. Tre, Suc, raffinose family oligosaccharides and fructans are involved in plant stress responses (Krasensky and Jonak, 2012; Keunen et al., 2013). Little is known about their role in autophagy, but it can be speculated that extreme accumulation may also lead to autophagy induction to prevent excessive build up. Intriguingly, maltose, a breakdown product of starch has been linked to SnRK1 activation, which in turn can activate autophagy to recycle carbon derived from starch breakdown during periods of stress (Wang et al., 2013; Ruiz-Gayosso et al., 2018).

Contrary to Arabidopsis, where increased trehalase (decreased endogenous Tre) resulted in increased drought tolerance (Van Houtte et al., 2013), increased Tre is known to promote desiccation tolerance in the Tre-accumulating resurrection species *Sporobolus stapfianus* (Gaff et al., 2009; Griffiths et al., 2014). Tre was suggested to induce autophagy (Williams et al., 2015), with possible involvement of SnRK1 (Asami et al., 2018). Interestingly, non-Tre accumulating resurrection species such as *Haberlea rhodopensis* stays green in prolonged darkness for several months, and SnRK1 seems to be a key player (Durgud et al., 2018).

## Concluding Remarks

Autophagy plays an important role to recycle cytosolic material and maintain cellular homeostasis during periods of stress, but also during the process of growth. The interplay between sugar signaling -and -autophagy-pathways in plants is complex and depends to a large extend on the organism and tissue type. SnRK1 and TOR contribute to the major energy and/or stress dependent regulation of autophagy; however, new advances suggest that alternative pathways also exist. Through SnRK1 and TOR, autophagy is regulated by sugar availability to recycle and provide the required resources for growth and development, and in turn autophagy assists in the removal of excess sugar from the cytosol, thus regulating the level of sugars available. This shows that sugars are not only important in the regulation of autophagy, but autophagy can also be important in regulating sugar homeostasis. Active TOR seems to be an overriding factor in the control of autophagy through energy-dependent pathways. Besides the SnRK1/TOR pathways, regulation of autophagy has also been linked to the G-protein signaling pathway in response to external Glc. The exact mechanisms and sequence of events need further investigation to understand whether this links to the SnRK1/TOR or TOR autophagy pathways or functions independently. It is also tempting to speculate that other SnRK complexes such as SnRK2 might regulate autophagy through ABA signaling pathways under stress. The major constrains in understanding sugar signaling and the interplay with autophagy is the complexity and variation of these pathways between sink and source tissues. In this regard it is important to take caution when comparing results between different species, organs or even growth stages.

## Author Contributions

All authors listed have made a substantial, direct and intellectual contribution to the work, and approved it for publication.

## Conflict of Interest Statement

The authors declare that the research was conducted in the absence of any commercial or financial relationships that could be construed as a potential conflict of interest.
